# Red-Mediated Transposition and Final Release of the Mini-F Vector of a Cloned Infectious Herpesvirus Genome

**DOI:** 10.1371/journal.pone.0008178

**Published:** 2009-12-04

**Authors:** Felix Wussow, Helmut Fickenscher, B. Karsten Tischer

**Affiliations:** 1 Institute for Infection Medicine, Christian-Albrecht University of Kiel and University Medical Center Schleswig-Holstein, Kiel, Germany; 2 Institute of Virology, Freie Universität Berlin, Berlin, Germany; Yale University, United States of America

## Abstract

Bacterial artificial chromosomes (BACs) are well-established cloning vehicles for functional genomics and for constructing targeting vectors and infectious viral DNA clones. Red-recombination-based mutagenesis techniques have enabled the manipulation of BACs in *Escherichia coli* without any remaining operational sequences. Here, we describe that the F-factor-derived vector sequences can be inserted into a novel position and seamlessly removed from the present location of the BAC-cloned DNA via synchronous Red-recombination in *E. coli* in an *en passant* mutagenesis-based procedure. Using this technique, the mini-F elements of a cloned infectious varicella zoster virus (VZV) genome were specifically transposed into novel positions distributed over the viral DNA to generate six different BAC variants. In comparison to the other constructs, a BAC variant with mini-F sequences directly inserted into the junction of the genomic termini resulted in highly efficient viral DNA replication-mediated spontaneous vector excision upon virus reconstitution in transfected VZV-permissive eukaryotic cells. Moreover, the derived vector-free recombinant progeny exhibited virtually indistinguishable genome properties and replication kinetics to the wild-type virus. Thus, a sequence-independent, efficient, and easy-to-apply mini-F vector transposition procedure eliminates the last hurdle to perform virtually any kind of imaginable targeted BAC modifications in *E. coli*. The herpesviral terminal genomic junction was identified as an optimal mini-F vector integration site for the construction of an infectious BAC, which allows the rapid generation of mutant virus without any unwanted secondary genome alterations. The novel mini-F transposition technique can be a valuable tool to optimize, repair or restructure other established BACs as well and may facilitate the development of gene therapy or vaccine vectors.

## Introduction

Bacterial artificial chromosomes (BACs) are low-copy F-factor derived replicon units and well-established vectors to clone large DNA molecules in *E. coli* [1], [2]. Regulatory elements of the mini-F replicon strictly maintain a BAC in one or maximally two copies per bacterial cell, reducing the potential for inter- and intramolecular homologous recombination between repeated sequences. Mini-F replicons can provide the stable propagation of complex DNA molecules up to 300 kb in recombination-deficient *E. coli* strains [1], [2]. Consequently, BACs are widely used in sequencing projects and functional genomics of diverse organisms, and for the construction of targeting vectors and infectious viral DNA clones [2]–[8]. However, the chimeric constitution of prokaryotic and eukaryotic sequences in addition to the current localization of the mini-F vector within the cloned DNA limits the versatility of an established BAC clone.

In the recent years, a number of recombination-based mutagenesis techniques have been introduced for rapidly manipulating large BAC-cloned DNA fragments in *E. coli* without leaving behind any operational elements [9]–[12]. Two-step *en passant* mutagenesis is a versatile and highly efficient markerless BAC manipulation technique, combining Red recombination with the cleavage by the homing endonuclease I-SceI [11], [13], [14]. In this procedure, large primers are designed to generate a PCR product for the insertion by Red recombination of a selection marker together with an I-SceI recognition site flanked by a 50 bp direct sequence duplication of the cloned DNA into the target sequence. Upon selection of recombinants, a double-strand break can be initiated at the I-SceI site to seamlessly excise the positive selection marker by a second Red-mediated recombination via the short duplicated sequences. Using appropriate plasmids and bacterial strains, *en passant* mutagenesis can be applied to rapidly and seamlessly generate point mutations or deletions and to insert large functional sequences [11], [15].

In order to facilitate the generation of mutant virus progeny, the large viral genomes of numerous herpesviruses and of some other viruses have been constructed in the past years as infectious BACs [16]–[22]. Full-length viral DNA can be maintained and seamlessly manipulated in *E. coli* and subsequently delivered into virus-permissive cells to allow the reconstitution of mutant progeny that will represent a uniform and homogenous virus population [16]. To investigate the neurotropic human α-herpesvirus varicella zoster virus (VZV), the causative agent of chickenpox and shingles [23], we previously constructed the 125 kb linear DNA genome of the European VZV wild-type strain HJO as infectious BAC (Schmidt M, and Fickenscher H, in preparation) [24]–[26].

The BAC technology has been proven to be a safe, fast, and effective approach to get insight into herpesvirus biology and is predicted to facilitate the development of therapeutic gene and vaccine vectors [3], [8]. To prevent interference of the mini-F elements with the viral functions, herpesvirus BACs are usually engineered in a way that the vector sequences can be removed upon virus reconstitution by site-specific recombinases, such as Cre, which leave behind only one residual small recognition site [27]. Complete removal of the bacterial elements during the virus reconstitution from an infectious BAC has been established by homologous recombination of engineered large genomic duplications [28], [29]. However, this strategy can be associated with illegitimate recombination events during the bacterial or viral DNA replication or it's efficiency can highly depend on the vector insertion site within the viral genome.

In this study, we demonstrate a novel technique based on *en passant* mutagenesis to seamlessly transpose the mini-F sequences within BAC-cloned DNA in *E. coli*. We developed this method to rapidly identify an optimal integration site for the mini-F sequences within the cloned genome of the VZV strain HJO, to induce their efficient and seamless excision from the viral DNA upon virus reconstitution in VZV-permissive cells. The insertion of the mini-F sequences directly between the VZV genomic termini resulted in a BAC variant of which the vector elements were rapidly released during the viral DNA replication even without additional operational sequences such as *loxP* sites or genomic duplications.

## Results

### Red-Recombination-Mediated Mini-F Vector Transposition

For the targeted and seamless transposition of the mini-F sequences of BAC-cloned DNA in *E. coli*, a second mini-F vector had to be inserted into a different position while removing the pre-existing mini-F cassette by two events of recombination. Since maximally two copies of a BAC can exist per bacterial cell [1], [2], we assumed that these reactions must be induced synchronously to prevent the uneven segregation or disruption of putative intermediates with two mini-F replicons during the strictly coordinated chromosomal-like proliferation upon bacterial cell division. Therefore, we developed a novel procedure to induce the targeted insertion of an additional mini-F vector into a novel position of the cloned DNA by a first Red recombination [14], simultaneously with the seamless removal of the primary mini-F cassette by an *en passant* mutagenesis-mediated second Red recombination [11], [15].

**Figure 1:**
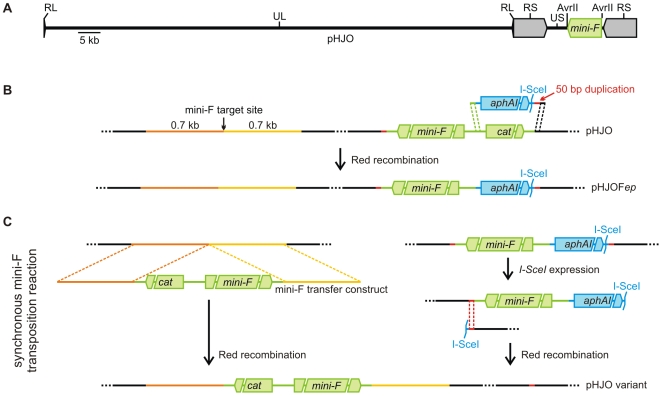
Mini-F vector transposition. **A**) The infectious BAC pHJO. In the pHJO BAC clone the mini-F sequences are located within the unique AvrII site of the VZV US region. RL: repeat of the long region; UL: unique region long; RS: repeat of the short region; US: unique region short. **B**) Preparation of the BAC for the mini-F transposition. An *aphAI*-I-SceI cassette (blue) was amplified with primers bearing specific 50 bp extensions (green and black). One primer provides a 50 bp duplication (red). The generated PCR product was applied to replace the *cat* gene of the pBeloBAC11 vector sequences (green) from pHJO, resulting in pHJOF*ep*. **C**) Synchronous mini-F transposition reaction. A mini-F transfer construct was used to insert a second pBeloBAC11 vector via Red-mediated recombination of flanking 0.7 kb homologous sequences (orange and yellow) into the target position of interest. After introduction of a double-strand break by I-SceI expression, the primary mini-F cassette was seamlessly excised by a second Red-mediated recombination of the short 50 bp duplication, resulting in a pHJO variant.

The original HJO BAC, termed pHJO, consists of the full-length VZV genome, which is organized into a unique long (UL) and a unique short (US) region, flanked by very small (RL) or large inverted (RS) repeats, respectively [30], and pBeloBAC11 vector elements within the unique AvrII site of the US region (Fig. 1A). In the first step of the *en passant* mutagenesis, pHJO was prepared for the mini-F sequence transposition reaction as follows (Fig. 1B). First, the kanamycin resistance gene *aphAI* and the homing endonuclease I-SceI restriction site of plasmid pEPkan-S [11] (Fig. 1B, blue elements) were amplified with primers bearing short extensions. One primer contained at the 5′ terminus 50 bp that were homologous to sequences at the border of the chloramphenicol resistance gene *cat* and the mini-F replicon of the pBeloBAC11 vector (Fig. 1B, green extension). The other primer consisted at the 5′ terminus of 50 bp that were homologous to HJO sequences directly adjacent to the *cat* gene of the pBeloBAC11 vector in pHJO (Fig. 1B, black extension). The latter primer provided also a 50 bp duplication homologous to viral sequences found adjacent to the mini-F replicon at the opposite pBeloBAC11 vector end in pHJO (Fig. 1B, red extension). Subsequently, the resulting PCR product was used to substitute the *cat* gene at one pBeloBAC11 vector end of pHJO via Red recombination by the *aphAI*-I-SceI cassette and the 50 bp homologous to viral sequence at the opposite vector end. The resulting BAC, termed pHJOF*ep*, contained an *aphAI*-I-SceI-associated mini-F replicon flanked by a 50 bp direct viral sequence duplication (Fig. 1B). Consequently, pHJOF*ep* was ready for the insertion of a second pBeloBAC11 vector into a target position of interest by Red recombination and selection for chloramphenicol resistance, and for the seamless excision of the *aphAI*-I-SceI-mini-F cassette by the second *en passant* mutagenesis step.

For the insertion of the pBeloBAC11 vector into the target position of interest a mini-F transfer constructs was generated using the plasmid pCeu2 [29]. This transfer construct consisted of a 6.4 kb modified pBeloBAC11 vector without *cos* and *loxP* sequences flanked by 0.7 kb viral sequences homologous to regions located at either site of the target position of interest (Fig. 1B and 1C, orange and yellow elements). The transfer construct was engineered within the multiple cloning site (*MCS*) between the two I-CeuI homing endonuclease restriction sites of plasmid pCeu2. To additionally avoid the degradation of the BAC in the following transposition procedure, the pBeloBAC11 vector elements were engineered in inverse orientation between the viral sequences with respect to the present mini-F fragment of pHJOF*ep* (Fig. 1B and 1C).

In the mini-F sequence transposition reaction, the pBeloBAC11 vector was inserted into the target position of interest using the prepared transfer construct and the *aphAI*-I-SceI-mini-F cassette was excised via two successively induced Red recombinations (Fig. 1C). First, the mini-F transfer construct was released from the pCeu2 vector backbone and used to insert the 6.4 kb pBeloBAC11 derivate via Red-mediated recombination of the 0.7 kb flanking homologous viral sequences into the target position of interest in pHJOF*ep* (Fig. 1C). Immediately after that, a double-strand break was introduced between the *aphAI* gene and the 50 bp duplication by *I-sceI* expression and the *aphAI*-mini-F cassette was seamlessly removed in the second *en passant* step by Red-mediated recombination of the short 50 bp duplication (Fig. 1C). To maintain the cloned HJO genome during this transposition reaction, the Red-mediated pBeloBAC11 insertion, the I-SceI-mediated double-strand break introduction, and the Red-mediated *aphAI*-mini-F fragment excision was induced in a novel synchronous reaction (for detailed protocol see Materials and Methods). The resulting BAC variant of pHJO with pBeloBAC11 vector in the target position of interest comprised a repaired unique AvrII site of the VZV US region (Fig. 1C).

### Versatility and Efficiency of the Mini-F Vector Transposition

**Figure 2:**
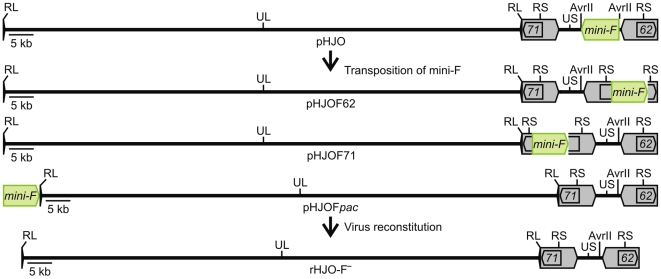
Infectious BAC clones with genome-intrinsic releasable mini-F sequences. Scheme of the generation of pHJO variants with mini-F sequences (green) releasable by intrinsic genome replication processes. Upon mini-F transposition in *E. coli*, the vector sequences were shifted within the BAC-cloned HJO genome (pHJO) from the unique AvrII site of the US region (reverse with respect to UL, regarding the defined VZV prototype genome organization) into *ORF62*, *ORF71*, or directly into genomic terminal junction of VZV, to generate pHJOF62, pHJOF71, or pHJOF*pac*, respectively. Virus reconstitution from the pHJO variants yielded vector-free progeny rHJO-F^-^.

Using corresponding mini-F transfer constructs, the pBeloBAC11 elements were transposed from the unique AvrII site of the VZV US region within the BAC-cloned HJO genome into *ORF22*, *ORF50*, or *ORF54* of the UL region, into either the coding domain of the duplicated *ORF62/71* of the large S repeats, or directly between the genomic termini. The resulting BACs were termed pHJOF22, pHJOF50, or pHJOF54 (Fig. S1 and S2), and pHJOF62, pHJOF71, or pHJOF*pac* (Fig. 2 and S2), respectively. Potential BAC recombinants with transposed pBeloBAC11 sequences were first identified by colony-PCR screening for determination of the mini-F excision from the unique AvrII site of the US region (Table 1).

**Table 1:** Efficiency of the mini-F vector transposition in *E. coli* and the vector self-excision upon virus reconstitution.

Efficiency of the mini-F transposition and the mini-F self-excision						
target	bacteria positive/tested			virus passage	viruses positive/tested	
	mini-F excision^a^	mini-F transposition^b^		mini-F detection^d^	mini-F excision^f^	
	Colony-PCR	RFLP	sequencing	PCR (experiments)	PCR	sequencing
*ORF22*	3/23	2/3	2/2^c^	4 (6)	6/6	2/2
*ORF50*	15/23	8/8	6/6^c^	4 (4)	4/4	2/2
*ORF54*	15/23	8/8	6/6^c^	4 (1)	1/1	1/1
*ORF62*	5/96	2/5	2/2	4; >7^e^ (2)	1/2^e^	1/1
*ORF71*	8/96	2/8	2/2	5; 6 (2)	2/2	2/2
Junction	9/168	3/9	1/1	2 (8)	8/8	16/16

a Colony-PCR screening for determination of the mini-F excision from the US unique AvrII site.

b Confirmation of the mini-F transposition into the given target site by RFLP and PCR or sequencing analysis.

c Only the repair of the unique AvrII site was investigated via sequencing.

d Average number of the last virus passage in which the mini-F sequences were detected by PCR after virus reconstitution. Numbers of the performed independent transfection experiments are given in brackets.

e Detection of mini-F until passage 7 and beyond.

f Confirmation of the mini-F excision in derived virus progenies by PCR or sequencing.

For example, upon mini-F transposition into *ORF50*, 15 of 23 tested colonies were positive by PCR screening for the mini-F sequence removal from the unique US AvrII site. Eight of the positive clones were tested by restriction fragment length polymorphism (RFLP) and PCR analysis, and the pBeloBAC11 transposition was confirmed in all these BACs. Six of the RFLP pattern-positive BACs were investigated via sequencing. The seamless repair of the unique AvrII site was confirmed in all BAC clones (Table 1). When transposing the pBeloBAC11 sequences into the terminal genomic junction, only 9 of 168 tested colonies were positive for the vector excision from the US AvrII site by PCR screening. In 3 of those 9 BACs the pBeloBAC11 transposition into the terminal genomic junction was confirmed by RFLP and PCR. The correct RFLP pattern was analyzed via sequencing, confirming the precise insertion of the pBeloBAC11 vector between the VZV DNA ends and the seamless repair of the US unique AvrII site (Table 1).

In conclusion, the pBeloBAC11 sequences were successfully transposed into specific target positions distributed over the unique or repeat regions of the VZV genome, to generate six different BAC variants of pHJO. Since the mini-F vector transposition into *ORF22* was less effective than into *ORF50* or *ORF54* (Table 1), the efficiency of the reaction seemed to decrease the more distant the target position of interest is located from the primary mini-F insertion site. It appeared also, that the mini-F transposition into positions of the unique regions is more efficient than into the repeated regions of the VZV genome. However, potential BAC candidates with transposed mini-F sequences, independently from in which position they were moved within the cloned VZV genome, can be rapidly identified via colony-PCR screening.

### Efficient Genome-Intrinsic Mini-F Vector Self-Excision from the VZV DNA Ends

In the nucleus of an infected cell, herpesviral DNA circularizes and replicates into branched and linear concatemeric molecules of serially and covalently linked genomic units [31], [32]. The concatemeric genome multimers are cleaved precisely at the junctions of the genomic termini by a terminase complex and the derived linear monomers are packed by a head-full sensing mechanism into preformed nascent particles [33], [34]. During DNA replication, sequence inversions or deletions can be mediated by inter- and intramolecular homologous recombination of inversely or directly repeated elements [35]–[37].

The pBeloBAC11 sequences were inserted into *ORF22*, *ORF50*, or *ORF54* to generate BAC variants comprising self-excisable vector elements with inverse genomic duplications in genes which are putatively essential or important for the virus propagation *in vitro*, but not for the viral DNA replication (Fig. S1; pHJOF22-DX, pHJOF50-DX, or pHJOF54-DX) [38]–[40]. This should induce the immediate vector elimination upon repair of the gene function by two events of homologous recombination of the genomic duplication as described previously [29]. Upon virus reconstitution from the three pHJO-DX variants in VZV-permissive MeWo melanoma cells, the vector sequences were seamlessly released from the viral DNA during four rounds of virus propagation (Table 1). However, in comparison to the virus reconstitution from the original pHJO BAC, the virus reconstitution from the pHJO-DX variants occurred only after one further cell passage of the transfected MeWo cells.

The single deletion of the duplicated *ORF62/71* of the large VZV S repeats was shown to lead to efficient repair of the disrupted repeat region by homologous recombination events with the intact repeat region [41]. Furthermore, it was surmised that the genomic termini are formed to some degree independently by two precise cleavages of a terminase complex [42]–[44]. In addition, sequences at the genomic termini were believed to be recombinational hot-spots due to the induction of an additional double-strand break mechanism or due to the high GC content at the DNA ends [35]–[37]. Therefore, we assumed that mini-F sequences inserted into one of the coding domains (*ORF62* or *ORF71*) of the immediate-early transactivator IE62 or into the terminal genomic junction can be rapidly released by intrinsic DNA replication or genome maturation processes, even without a genomic duplication. For this reason, we transposed the mini-F sequences into *ORF62*, *ORF71* or directly between the VZV DNA ends, to generate pHJOF62, pHJOF71 or pHJOF*pac*, respectively (Fig. 2). These BACs could stably be propagated in *E. coli*, since unexpected rearrangements in comparison to the mini-F excision construct pHJOF*ep* and the original pHJO BAC were not detected by RFLP analysis (Fig. S2).

Virus reconstitution by the detection of the VZV-typical focal plaques from pHJOF62 and pHJOF71 upon transfection into MeWo cells was observed after four to five days, which corresponds with the time period required for virus recovery from the original pHJO BAC. Remaining pBeloBAC11 elements were detected in virus progeny recovered from two pHJOF62 and pHJOF71 BAC clones until virus passage four or seven - and beyond - and five or six respectively (Table 1, data not shown). The seamless repair of *ORF62* or *ORF71* was confirmed in amplified PCR fragments from the repeat regions of each of the vector-free progenies generated from the different BAC variants (Table 1, data not shown).

**Figure 3:**
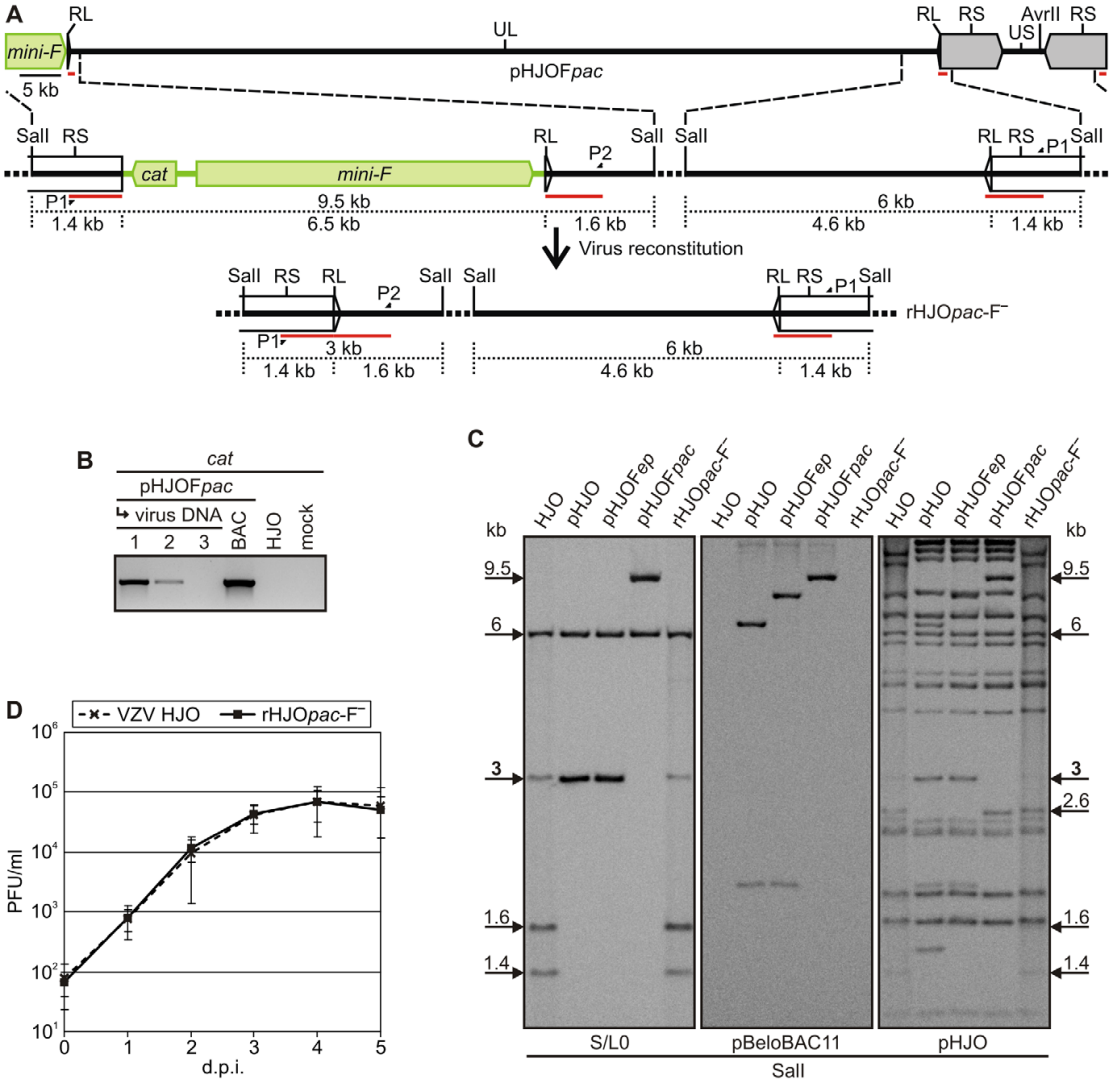
Mini-F sequence transposition into the genomic terminal junction and release upon virus reconstitution. **A**) Detailed overview of the genomic transitions in pHJOF*pac* and rHJO*pac*-F^-^. The vector sequences (green) were released upon virus reconstitution from pHJOF*pac* and vector-free progeny rHJO*pac*-F^-^ was generated. Lengths of both transitional SalI fragments and the distances from the SalI restriction sites to the genomic junctions are given (black dotted lines). Also, the binding sites of the Southern-blot probe (red bars) for analyzing the terminal junction and of the primers P1 and P2 (Table S1) used to generate the probe are indicated. **B**) PCR analysis of the mini-F sequence release upon virus reconstitution. Total DNA from MeWo cells infected with pHJOF*pac*-derived progeny of virus passage 1–3 was analyzed with the *cat*-specific primers P3 and P4 (Table S1). BAC DNA of original pHJO as well as total DNA from MeWo cells infected with HJO wild-type or uninfected cells were analyzed as controls. **C**) Southern-blot analysis of pHJOF*pac* and rHJO*pac*-F^-^ in comparison to HJO, pHJO, pHJOF*ep*. BAC DNA was prepared from *E. coli* harboring pHJO, pHJOF*ep*, or pHJOF*pac*. Total DNA from MeWo cells infected with HJO or rHJO*pac*-F^-^ was purified for examination of virus DNA. The isolated DNA was digested with SalI, separated electrophoretically on a 1% agarose gel, and blotted. Genomic junction-, vector-, or VZV sequences were detected with probes specific for viral sequences spanning the genomic transition at the *ORF0* end of the UL region (termed S/L0, red bars in A), the pBeloBAC11 plasmid, or pHJO DNA, respectively. The 3 kb or 6 kb SalI fragments corresponding to the terminal or internal junction as well as the 9.5 kb SalI fragments that correspond to the terminal junction with mini-F vector in pHJOF*pac* as presented in A are marked. Also indicated are the 1.6 kb and 1.4 kb SalI fragments that correspond to termini of linear genomes within viral DNA as outlined in A as well as the 2.6 kb SalI fragments that correspond to the restored US unique AvrII site (arrows flanking the picture frame). **D**) Growth kinetics of rHJO*pac*-F^-^ and HJO. Multistep growth kinetics of rHJO*pac*-F^-^ compared to wild-type virus HJO in MeWo cell cultures was performed. Means and standard deviations of two independent experiments with three separate results each are given.

Virus reconstitution by the generation of the VZV-typical plaques was also observed after four to five days upon transfection of MeWo cells with pHJOF*pac*. In the derived progeny, the vector elements were only traceable until virus passage two (Fig. 3B). This observation could be confirmed by eight independent transfection experiments (Table 1). Thus, the pHJOF*pac* construct appeared to be the ideal candidate to rapidly generate vector-free recombinant progeny. Therefore, the pHJOF*pac*-derived virus was analyzed in detail.

**Figure 4:**
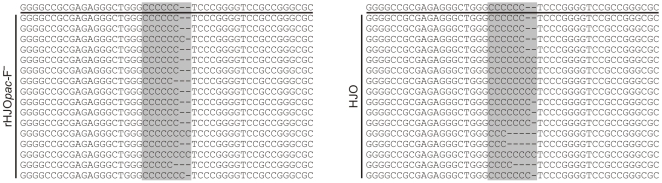
Sequence analysis after mini-F vector release from the genomic termini. Sequence analysis of 16 cloned PCR fragments that were generated by PCR amplification of the terminal genomic junction of rHJO*pac*-F^-^ or wild-type HJO DNA. The derived sequences were aligned to the sequence of the terminal transition of the original pHJO clone (first line). The GC tracts directly at the junction are highlighted in gray.

Due to nucleotide polymorphisms at the VZV DNA ends, PCR products amplified from the terminal junction of pHJOF*pac*-derived mini-F sequence-free virus rHJO*pac*-F^-^ as well as from wild-type virus as a positive control were first cloned and then sequenced to determine the seamless mini-F vector excision. The repair of the terminal junction was confirmed in 16 cloned PCR fragments derived from rHJO*pac*-F^-^ (Fig. 4). A variability of the number of the GC base pairs (ranging from 3–8) at the junction was detected in DNA of rHJO*pac*-F^-^ and HJO wild-type virus (Fig. 4).

Subsequently, the genome of rHJO*pac*-F^-^ was investigated via Southern-blot analysis, at the terminal junction, for complete integrity, and for the absence of any pBeloBAC11 sequences. The restriction fragment patterns were confirmed to be indistinguishable from that of wild-type HJO DNA and pBeloBAC11 sequences were only detected in pHJO, pHJOF*ep* and pHJOF*pac* BAC DNA, whereas not in the viral DNA (Fig. 3C). Finally, the replication kinetics of rHJO*pac*-F^-^ upon propagation in MeWo cells was determined. The obtained growth curve of the recombinant virus was comparable to that of wild-type virus HJO (Fig. 3D). Thus, pHJOF*pac* enables the rapid generation of recombinant progeny that has genome and growth properties virtually identical to the parental HJO virus. In conclusion, the mini-F sequences were seamlessly released from the genomic termini by intrinsic features of the VZV DNA replication process and, in addition, the integrity of the complete HJO genome was conserved during the entire BAC cloning and mini-F transposition procedure in *E. coli*.

## Discussion

In this work, we demonstrated that the low-copy F-factor sequences can be targeted and seamlessly transposed within BAC-cloned DNA in *E. coli* via Red recombination. We also demonstrated that the mini-F vector of an infectious herpesvirus BAC can be efficiently and seamlessly released from the genomic termini upon virus reconstitution in eukaryotic cells by inherent genome replication or maturation processes.

By *en passant* mutagenesis [11], [15], it was possible to induce the vector insertion into a target position of interest and the vector removal from the present integration site via two synchronous Red recombinations combined with an interposed I-SceI-mediated double-strand break (Fig. 1C). The versatility and efficiency of the developed transposition technique was proved by shifting the mini-F vector into different unique and repeated regions distributed over a cloned herpesvirus genome, finally resulting in six BAC variants (Fig. 2 and S1). It appears therefore that the mini-F transposition reaction enables the rapid generation of virtually every specific variant of an established BAC in *E. coli* under perfect preservation of the integrity of the cloned DNA. In the context of the recently introduced highly versatile and markerless recombineering techniques [9]–[12], [15], this method eliminates the last hurdle to perform virtually any kinds of imaginable targeted BAC modifications. The mini-F transposition reaction can be therefore generally a valuable tool to optimize, reorganize, or repair other established BACs as well, which may have an immediate impact on the development of gene therapy or vaccine vectors [3], [6], [8]. Also BACs generated by random cloning techniques could be adapted subsequently by transposition of the mini-F vector to a defined position to tweak those clones for further work [45]. In addition this may be also useful to facilitate the generation of optimized targeting vectors for mutant animals, such as conditional knock-out mice [2], [4], [5], [7].

The targeted generation of an infectious herpesvirus BAC includes recombination during the virus propagation in eukaryotic cells and transfer of circular replication intermediates into *E. coli* [16], [29]. Therefore, it is difficult to integrate the mini-F vector into repeated regions with self-repair potential or into sites involved in the viral genome replication or maturation process. Using the mini-F transposition method, we developed the first herpesvirus BACs (Fig. 2), of which the vector sequences were efficiently released by intrinsic features during the genome maturation process, thus, without the need of additional sequences, such as *loxP* sites or a genomic duplication [27]–[29]. We identified the herpesviral terminal genomic junction as an optimal mini-F vector integration site for the construction of an infectious BAC that provides the rapid and spontaneous generation of recombinant progeny that is indistinguishable to the wild-type virus *in vitro* (Fig. 3 and 4). This strategy to generate mutant herpesvirus progeny does not only avoid the remaining of any vector sequences in the viral genome it also reduces the risk of illegitimate events of homologous recombination of the chimeric DNA during the bacterial or viral DNA replication. The terminal genomic junction might therefore be in general an optimal integration site of the mini-F sequences to construct large linear DNA genomes as infectious herpesvirus BACs.

The delayed virus reconstitution from the three pHJO-DX variants (Fig. S1) may correspond with the time period required for the mini-F excision to repair *ORF22*, *ORF50*, or *ORF54* by homologous recombination of the genomic duplications [29]. However, despite of the introduction of a highly detrimental effect on the virus replication by the mini-F sequences within the ORFs, it was unexpected that remaining pBeloBAC11 sequences were detected in three further passages after virus reconstitution (data not shown). This phenomenon may be explained by the highly cell-associated growth of VZV, which may cause an intimate polyclonal virus spread from cell to cell and, thus, allow the propagation of remaining pBeloBAC11 sequences upon *trans*-complementation [23], [46], [47]. In conclusion, the three BACs, pHJOF22-DX, pHJOF50-DX, and pHJOF54-DX (Fig. S1), allow the efficient and seamless vector release upon virus reconstitution, but appear to be suboptimal for further mutant generation, because the virus reconstitution occurs at delayed kinetics.

The vector release in virus recovered from pHJOF62 or pHJOF71 (Fig. 2) may indicate that *ORF62* or *ORF71* of the large S repeats were efficiently repaired by inherent homologous recombination events during the viral DNA replication (Fig. S3) [41]. To exclude any illegitimate recombination upon the vector elimination from the repeat regions, the genome organizations of the derived viruses had to be analyzed for instance by Southern-blot hybridization. Nevertheless, the BAC variants allowed the virus reconstitution in eukaryotic cells and the vector elements were released upon propagation of the recombinant progeny, accepting them as valuable alternatives to generate vector-free mutant VZV.

The observation that the mini-F sequences in progeny reconstituted from pHJOF*pac* were much more efficiently released in comparison to that of the other generated recombinant viruses (Table 1), may support the proposal that the vector sequences can be actively excised from the genomic termini. This conclusion would then greatly support the assumption that the formation of the herpesvirus genomic termini is to some degree independently governed from the respective packaging signals [42]–[44]. Besides such an active cleavage mechanism, the mini-F vector excision from the genomic terminal junction may also be effectuated by highly frequent homologous recombination of the viral repeat elements, supported by a different double-strand break mechanism or by a high GC content [35], [37]. However, the VZV L repeats are only 88 bp in length [30], which makes an efficient intrinsic vector release unlikely that happens just by homologous recombination of the RL and RS regions flanking the mini-F cassette with the RL and RS regions of the intact junction (Fig. 3A).

The pHJO*pac* clone enables the rapid generation of virus progeny that is virtually identical in its genome and replication kinetics to wild-type virus HJO. This BAC variant has already been used successfully in bacteria to introduce further targeted mutations into the cloned viral genome and to derive vector-free VZV mutants in eukaryotic cell culture (Krater T, Brunnemann A, and Fickenscher H, unpublished). In conclusion, pHJOF*pac* is an ideal construct for the rapid production of VZV mutants without the retention of any unwanted operational sequences in the manipulated viral genome.

In summary, we have developed a method for the targeted and seamless restructuring of an established BAC in *E. coli*. Using this tool, we finally engineered a herpesvirus BAC with mini-F sequences between the genomic termini, which allows the rapid production of recombinant virus virtually identical to the wild-type.

## Materials and Methods

### Construction of the Transfer Vectors

To remove *loxP* and *cos* sequences and to introduce a BamHI or SphI site, pBeloBAC11 (Invitrogen, Karlsruhe, Germany) was digested with SalI and the released 6.4 kb fragment was ligated with the XhoI-treated hybrid of the oligonucleotides A and B or C and D (Table S1), to yield pBeloBamHI or pBeloSphI, respectively.

For the mini-F sequence transfer into *ORF22*, *ORF50*, *ORF54*, or *ORF62*/*71*, viral sequences were amplified with primers E and F, G and H, I and J or K and L (Table S1), and the 1.4 kb PCR products were inserted after SpeI digestion into the XbaI site of plasmid pCeu2 [29]. To generate a BamHI site for mini-F sequence transfer into the terminal junction, 0.7 kb PCR-fragments amplified from both genomic ends using primers M and N or O and P (Table S1), were digested with SalI or SpeI and successively inserted into the XhoI or XbaI site of pCeu2. Subsequently, the mini-F plasmid pBeloSphI was inserted into the SphI site of the cloned fragments of *ORF22* and *ORF54*, and pBeloBamHI was ligated into the BamHI site of the cloned fragments of *ORF50*, *ORF62*/*71* (in forward and reverse orientation) or the cloned genomic terminal transition. Hence, six different pBelo-HJO-in transfer plasmids were generated.

Transfer constructs for the insertion of a genomic duplication into the pBeloBAC11 sequences within *ORF22*, *ORF50*, or *ORF54* were generated by using the primers E and F, G and H or I and J. The SpeI-digested PCR products were inserted into the BlnI site of the plasmid pEP*MCS*-in-Belo [29], resulting in three different pEP-HJO-DX-in plasmids.

### Red Recombination

Red-mediated modification of BAC DNA was performed as described using the *E. coli* strain GS1783, which contains a temperature-dependent expression cassette for the recombination proteins and an L-(+)-arabinose-inducible *I-sceI* gene [11], [14], [15]. PCR products used for *en passant* mutagenesis were generated with polyacrylamide-gel electrophoresis-purified primers (Biomers, Ulm, Germany).

In the novel transposition reaction, *E. coli* GS1783 harboring pHJOF*ep* (Fig. 1B) were grown in the presence of kanamycin and prepared for Red recombination and electroporated as described before [11], [14]. Approximately 100–300 ng of a 7.8 kb mini-F transfer construct derived by I-CeuI-digestion of a corresponding pBelo-HJO-in plasmid were introduced by electroporation into GS1783 to insert the 6.4 kb pBeloBAC11 derivate via the 0.7 kb homologous sequences into the target position of interest in pHJOF*ep* (Fig. 1C). Afterwards, the bacteria were resuspended in 1 ml LB medium without antibiotics and grown for 2 h at 32°C. The second *en passant* step was induced to immediately remove the pre-existing pBeloBAC11 vector, simultaneously maintaining recombinant DNA with the newly inserted mini-F vector (Fig. 1C). Therefore, 1 ml pre-warmed LB medium with 60 µg/ml chloramphenicol and 1% arabinose was added to the suspension to select for *cat* co-integrates and to induce simultaneously a double-strand break at the I-SceI site adjacent to the *aphAI*-associated pBeloBAC11 mini-F cassette (Fig. 1C). Bacteria were grown for an additional hour at 32°C until shifted for 30 min to 42°C to express the Red proteins again, enabling the excision of the *aphAI*-associated pBeloBAC11 cassette by recombination of the flanking 50 bp viral sequence duplication (Fig. 1C). Finally, the suspension was grown for 2 h at 32°C and bacteria were selected at 32°C on agar plates containing 30 µg/ml chloramphenicol and 1% arabinose.

### Generation of the BAC Constructs

To generate pHJOF*ep*, an *aphAI*-I-SceI cassette from pEPkan-S [11] was amplified with the primers Q and R (Table S1), which provide a 50 bp viral sequence duplication, and the PCR product was used to replace *cat* from pHJO via Red recombination (Fig. 1B). For the generation of the different pHJO variants, the pBelo-HJO-in transfer constructs were digested with I-CeuI and the obtained 7.8 kb fragments were used in the transposition reaction (Fig. 1) to insert 6.4 kb pBeloBAC11 sequences into a novel position and to release the mini-F vector sequences of pHJOF*ep*, resulting in pHJOF22, pHJOF50, pHJOF54, pHJOF62, pHJOF71, or pHJOF*pac* (Fig. 2 and S1).

The genomic duplications were inserted into pHJOF22, pHJOF50, or pHJOF54 as described previously [29]. The corresponding transfer plasmid pEP-HJO-DX-in was digested with I-CeuI and the release 4.2 kb fragments were used in an *en passant* procedure to insert the viral sequence duplications in reverse orientation into the mini-F vector, resulting in pHJOF22-DX, pHJOF50-DX, or pHJOF54-DX (Fig. S1).

### Virus Cultivation

Viruses were propagated in permissive MeWo melanoma cells grown in *Dulbecco's Minimal Essential Medium* (DMEM) containing 10% heat-inactivated fetal bovine serum, penicillin, and streptomycin (PAA, Cölbe, Germany). Virus was passaged by co-cultivation of infected cells with uninfected MeWo cells derived from confluent monolayers to yield cultures with a CPE of 70–80% after 6 to 7 days. The complete sequence of the VZV strain HJO is available in GenBank under accession number AJ871403.1 [24], [25].

### Virus Reconstitution from BAC DNA

Virus reconstitution was achieved by transfection of MeWo cells with BAC DNA using Lipofectamine2000 (Invitrogen) according to the manufacturer's instructions. Transfection was performed in 6-well format with 4 µg of purified BAC DNA (Plasmid Maxi Kit, Qiagen, Hilden, Germany). DNA-lipofectamine complexes were added to the cells in DMEM without L-glutamine. Transfection medium was replaced after 24 h with normal culture medium.

### Southern-Blot Hybridization

Southern-blot hybridization was performed using the DIG High Prime DNA Labelling and Detection Starter Kit II (Roche, Mannheim, Germany) according to manufacturer's instructions. Probes specific for pHJO or pBeloBAC11 fragments were generated by random labelling with digoxigenin of sonicated purified BAC DNA. The probe specific for the terminal transition was derived from PCR products generated with primers P1 and P2 (Fig. 3A and Table S1) using HJO DNA as template. For analyzing viral DNA, total DNA of infected MeWo cells from cultures with CPE of 70–80% cells was purified with the DNeasy Blood & Tissue Kit (Qiagen) according to the manufacturer's recommendations. BAC DNA was prepared from *E. coli* by alkaline lysis. Viral DNA (approximately 10 µg) and BAC DNA (1–2 µg) was digested with restriction endonucleases, separated on a 1% agarose gel and blotted to a positively charged nylon membrane. Hybridizations were finally visualized by chemiluminescence detection using the LAS-3000 imaging system (Fuji, Tokyo, Japan)

### Multistep Virus Replication Kinetics

Replication kinetics of rHJO*pac*-F^-^ and HJO wild-type virus were determined as follows. First, 1×10^6^ MeWo cells were seeded in 6-well plates and inoculated after 24 h with 100 plaque- forming units of titrated and frozen cell-associated virus. Cells of three wells each were trypsinized on day 1 to 5 and separately titrated in quadruplicates by co-cultivation of serial 10-fold dilutions of the harvested inocula with subconfluent uninfected MeWo cells (2×10^5^ cells) seeded in 24-well plates the day before. Viral plaques were stained 3 days after infection by indirect immunofluorescence using a monoclonal antibody against glycoprotein I (anti-VZV-gI, Millipore, Schwalbach, Germany) and counted using the Olympus microscope IX81. The entire experiment was performed twice and growth curves were established by the average of 6 parallel results for each time point.

### Immunofluorescence

Cells were fixed with 2% paraformaldehyde in phosphate-buffered saline (PBS), permeabi-lized with 0.1% saponin in PBS, and blocked with 10% fetal bovine serum (FBS) in PBS. Cells were incubated with the primary antibody monoclonal anti-VZV-gI diluted 1∶500 in PBS containing 10% FBS and 0.02% sodium azide and subsequently with the secondary Alexa 488-conjugated goat anti-mouse antibody (Invitrogen) diluted 1∶1,000 in PBS with 10% FBS. Fluorescence was visualized with an Olympus IX81 microscope.

## Supporting Information

Figure S1 Construction of BAC variants with self-excisable mini-F vector in essential genes.
(0.12 MB PDF)

Figure S2 RFLP analysis of the mini-F vector transposition and insertion of genomic duplication.
(0.06 MB PDF)

Figure S3 Genome-intrinsic mini-F vector release from *ORF62/71*.
(0.05 MB PDF)

Table S1 Detection (A), Cloning (B) and *En passant* (C) primers used in this study.
(0.01 MB PDF)

## References

[1] Shizuya H, Birren B, Kim UJ, Mancino V, Slepak T (1992). Cloning and stable maintenance of 300-kilobase-pair fragments of human DNA in Escherichia coli using an F-factor-based vector.. Proc Natl Acad Sci U S A.

[2] Shizuya H, Kouros-Mehr H (2001). The development and applications of the bacterial artificial chromosome cloning system.. Keio J Med.

[3] Wagner M, Ruzsics Z, Koszinowski UH (2002). Herpesvirus genetics has come of age.. Trends Microbiol.

[4] Copeland NG, Jenkins NA, Court DL (2001). Recombineering: a powerful new tool for mouse functional genomics.. Nat Rev Genet.

[5] Yang XW, Gong S (2005). An overview on the generation of BAC transgenic mice for neuroscience research.. Curr Protoc Neurosci, Chapter.

[6] Sparwasser T, Eberl G (2007). BAC to immunology - bacterial artificial chromosome-mediated transgenesis for targeting of immune cells.. Immunology.

[7] Ristevski S (2005). Making better transgenic models: conditional, temporal, and spatial approaches.. Mol Biotechnol.

[8] Brune W, Messerle M, Koszinowski UH (2000). Forward with BACs: new tools for herpesvirus genomics.. Trends Genet.

[9] Sharan SK, Thomason LC, Kuznetsov SG, Court DL (2009). Recombineering: a homologous recombination-based method of genetic engineering.. Nat Protoc.

[10] Sawitzke JA, Thomason LC, Costantino N, Bubunenko M, Datta S (2007). Recombineering: in vivo genetic engineering in E. coli, S. enterica, and beyond.. Methods Enzymol.

[11] Tischer BK, von Einem J, Kaufer B, Osterrieder N (2006). Two-step red-mediated recombination for versatile high-efficiency markerless DNA manipulation in Escherichia coli.. Biotechniques.

[12] Warming S, Costantino N, Court DL, Jenkins NA, Copeland NG (2005). Simple and highly efficient BAC recombineering using galK selection.. Nucleic Acids Res.

[13] Murphy KC (1998). Use of bacteriophage lambda recombination functions to promote gene replacement in Escherichia coli.. J Bacteriol.

[14] Lee EC, Yu D, Martinez de Velasco J, Tessarollo L, Swing DA (2001). A highly efficient Escherichia coli-based chromosome engineering system adapted for recombinogenic targeting and subcloning of BAC DNA.. Genomics.

[15] Tischer BK, Smith G, Osterrieder N, Braman J (2009). En passant Mutagenesis - A two step markerless Red recombination system.. In Vitro Mutagenesis Protocols.

[16] Messerle M, Crnkovic I, Hammerschmidt W, Ziegler H, Koszinowski UH (1997). Cloning and mutagenesis of a herpesvirus genome as an infectious bacterial artificial chromosome.. Proc Natl Acad Sci U S A.

[17] Delecluse HJ, Hilsendegen T, Pich D, Zeidler R, Hammerschmidt W (1998). Propagation and recovery of intact, infectious Epstein-Barr virus from prokaryotic to human cells.. Proc Natl Acad Sci U S A.

[18] Stavropoulos TA, Strathdee CA (1998). An enhanced packaging system for helper-dependent herpes simplex virus vectors.. J Virol.

[19] Horsburgh BC, Hubinette MM, Tufaro F (1999). Genetic manipulation of herpes simplex virus using bacterial artificial chromosomes.. Methods Enzymol.

[20] Domi A, Moss B (2002). Cloning the vaccinia virus genome as a bacterial artificial chromosome in Escherichia coli and recovery of infectious virus in mammalian cells.. Proc Natl Acad Sci U S A.

[21] Cottingham MG, Andersen RF, Spencer AJ, Saurya S, Furze J (2008). Recombination-mediated genetic engineering of a bacterial artificial chromosome clone of modified vaccinia virus Ankara (MVA).. PLoS One.

[22] Almazan F, Gonzalez JM, Penzes Z, Izeta A, Calvo E (2000). Engineering the largest RNA virus genome as an infectious bacterial artificial chromosome.. Proc Natl Acad Sci U S A.

[23] Cohen JI, Straus SE, Arvin AM (2007). Varicella-zoster virus replication, pathogenesis, and management..

[24] Kress M, Fickenscher H (2001). Infection by human varicella-zoster virus confers norepinephrine sensitivity to sensory neurons from rat dorsal root ganglia.. FASEB J.

[25] Schmidt M, Kress M, Heinemann S, Fickenscher H (2003). Varicella-zoster virus isolates, but not the vaccine strain OKA, induce sensitivity to alpha-1 and beta-1 adrenergic stimulation of sensory neurones in culture.. J Med Virol.

[26] Loparev VN, Gonzalez A, eon-Carnes M, Tipples G, Fickenscher H (2004). Global identification of three major genotypes of varicella-zoster virus: longitudinal clustering and strategies for genotyping.. J Virol.

[27] Smith GA, Enquist LW (2000). A self-recombining bacterial artificial chromosome and its application for analysis of herpesvirus pathogenesis.. Proc Natl Acad Sci U S A.

[28] Wagner M, Jonjic S, Koszinowski UH, Messerle M (1999). Systematic excision of vector sequences from the BAC-cloned herpesvirus genome during virus reconstitution.. J Virol.

[29] Tischer BK, Kaufer BB, Sommer M, Wussow F, Arvin AM (2007). A self-excisable infectious bacterial artificial chromosome clone of varicella-zoster virus allows analysis of the essential tegument protein encoded by ORF9.. J Virol.

[30] Davison AJ, Scott JE (1986). The complete DNA sequence of varicella-zoster virus.. J Gen Virol.

[31] Ben-Porat T, Tokazewski SA (1977). Replication of herpesvirus DNA. II. Sedimentation characteristics of newly synthesized DNA.. Virology.

[32] Garber DA, Beverley SM, Coen DM (1993). Demonstration of circularization of herpes simplex virus DNA following infection using pulsed field gel electrophoresis.. Virology.

[33] Boehmer PE, Lehman IR (1997). Herpes simplex virus DNA replication.. Annu Rev Biochem.

[34] Boehmer PE, Nimonkar AV (2003). Herpes virus replication.. IUBMB Life.

[35] Thiry E, Meurens F, Muylkens B, McVoy M, Gogev S (2005). Recombination in alphaherpesviruses.. Rev Med Virol.

[36] McVoy MA, Ramnarain D (2000). Machinery to support genome segment inversion exists in a herpesvirus which does not naturally contain invertible elements.. J Virol.

[37] Umene K (1999). Mechanism and application of genetic recombination in herpesviruses.. Rev Med Virol.

[38] Desai PJ (2000). A null mutation in the UL36 gene of herpes simplex virus type 1 results in accumulation of unenveloped DNA-filled capsids in the cytoplasm of infected cells.. J Virol.

[39] Patel AH, Rixon FJ, Cunningham C, Davison AJ (1996). Isolation and characterization of herpes simplex virus type 1 mutants defective in the UL6 gene.. Virology.

[40] Yamagishi Y, Sadaoka T, Yoshii H, Somboonthum P, Imazawa T (2008). Varicella-zoster virus glycoprotein M homolog is glycosylated, is expressed on the viral envelope, and functions in virus cell-to-cell spread.. J Virol.

[41] Sato B, Ito H, Hinchliffe S, Sommer MH, Zerboni L (2003). Mutational analysis of open reading frames 62 and 71, encoding the varicella-zoster virus immediate-early transactivating protein, IE62, and effects on replication in vitro and in skin xenografts in the SCID-hu mouse in vivo.. J Virol.

[42] Wang JB, Nixon DE, McVoy MA (2008). Definition of the minimal cis-acting sequences necessary for genome maturation of the herpesvirus murine cytomegalovirus.. J Virol.

[43] Varmuza SL, Smiley JR (1985). Signals for site-specific cleavage of HSV DNA: maturation involves two separate cleavage events at sites distal to the recognition sequences.. Cell.

[44] Schynts F, McVoy MA, Meurens F, Detry B, Epstein AL (2003). The structures of bovine herpesvirus 1 virion and concatemeric DNA: implications for cleavage and packaging of herpesvirus genomes.. Virology.

[45] Zhou F, Li Q, Gao SJ (2009). A sequence-independent in vitro transposon-based strategy for efficient cloning of genomes of large DNA viruses as bacterial artificial chromosomes.. Nucleic Acids Res.

[46] Weller TH (1953). Serial propagation in vitro of agents producing inclusion bodies derived from varicella and herpes zoster.. Proc Soc Exp Biol Med.

[47] Chen JJ, Zhu Z, Gershon AA, Gershon MD (2004). Mannose 6-phosphate receptor dependence of varicella zoster virus infection in vitro and in the epidermis during varicella and zoster.. Cell.

