# Supplementary material for: Red-Mediated Transposition and Final Release of the Mini-F Vector of a Cloned Infectious Herpesvirus Genome
Source: PLoS One. 2009 Dec 4;4(12):e8178. doi: 10.1371/journal.pone.0008178 (PMC2780728; doi:10.1371/journal.pone.0008178)
Supplement: Figure S1 — Construction of BAC variants with self-excisable mini-F vector in essential genes. (0.12 MB PDF) [file pone.0008178.s001.pdf]

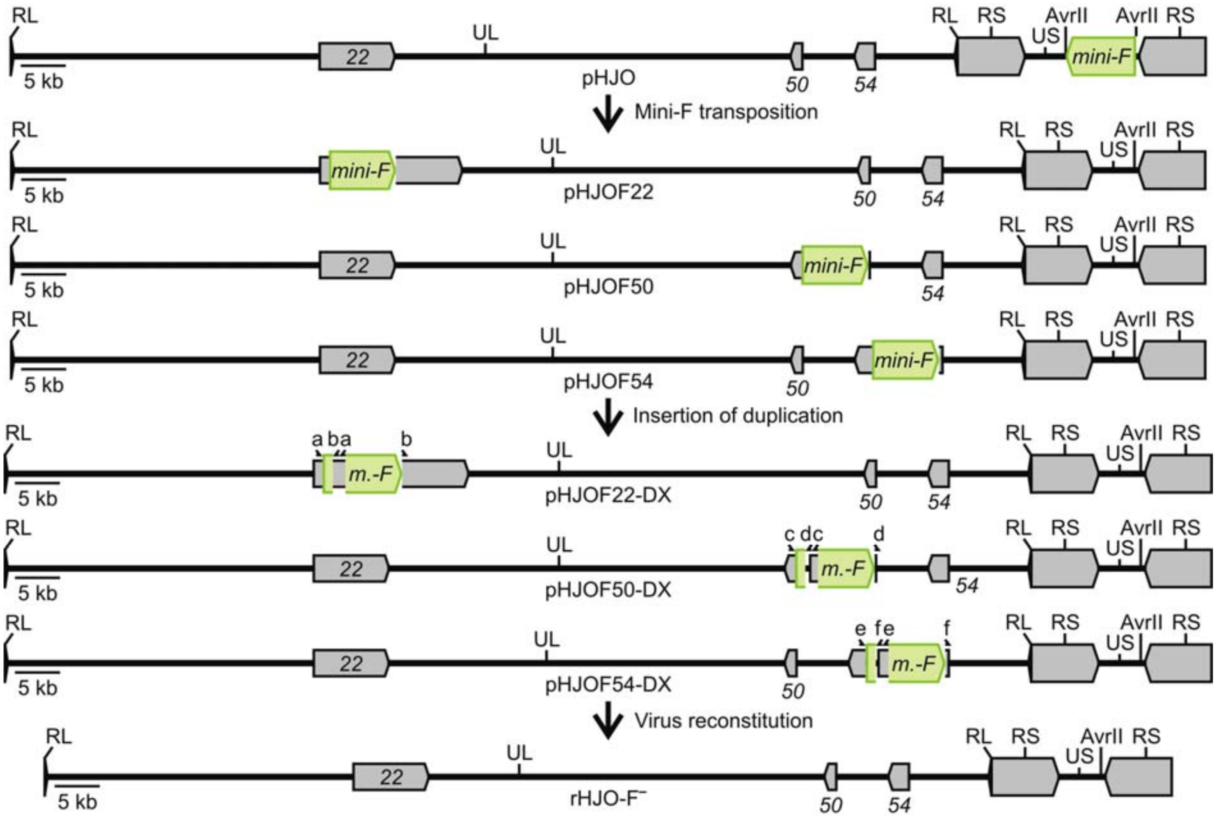


**Figure S1. Construction of BAC variants with self-excisable mini-F vector in essential genes.** First, the pBeloBAC11 vector (green) of pHJO was transposed from the unique AvrII site of the US region into *ORF22*, *ORF50,* or *ORF54*, resulting in pHJOF22, pHJOF50, or pHJOF54. Second, 1.4 kb genomic duplications of respective 0.7 kb viral sequences flanking the mini-F insertion site (a, b; c, d; e, f; sequence orientation by little arrows) were inserted in reverse orientation into the BAC vector to generate pHJOF22-DX, pHJOF50-DX, or pHJOF54-DX. Homologous recombination of the duplicated sequences upon virus reconstitution in transfected eukaryotic cells resulted in mini-F sequence-free virus rHJO-F$^-$.
