# Supplementary material for: Red-Mediated Transposition and Final Release of the Mini-F Vector of a Cloned Infectious Herpesvirus Genome
Source: PLoS One. 2009 Dec 4;4(12):e8178. doi: 10.1371/journal.pone.0008178 (PMC2780728; doi:10.1371/journal.pone.0008178)
Supplement: Figure S2 — RFLP analysis of the mini-F vector transposition and insertion of genomic duplication. (0.06 MB PDF) [file pone.0008178.s002.pdf]

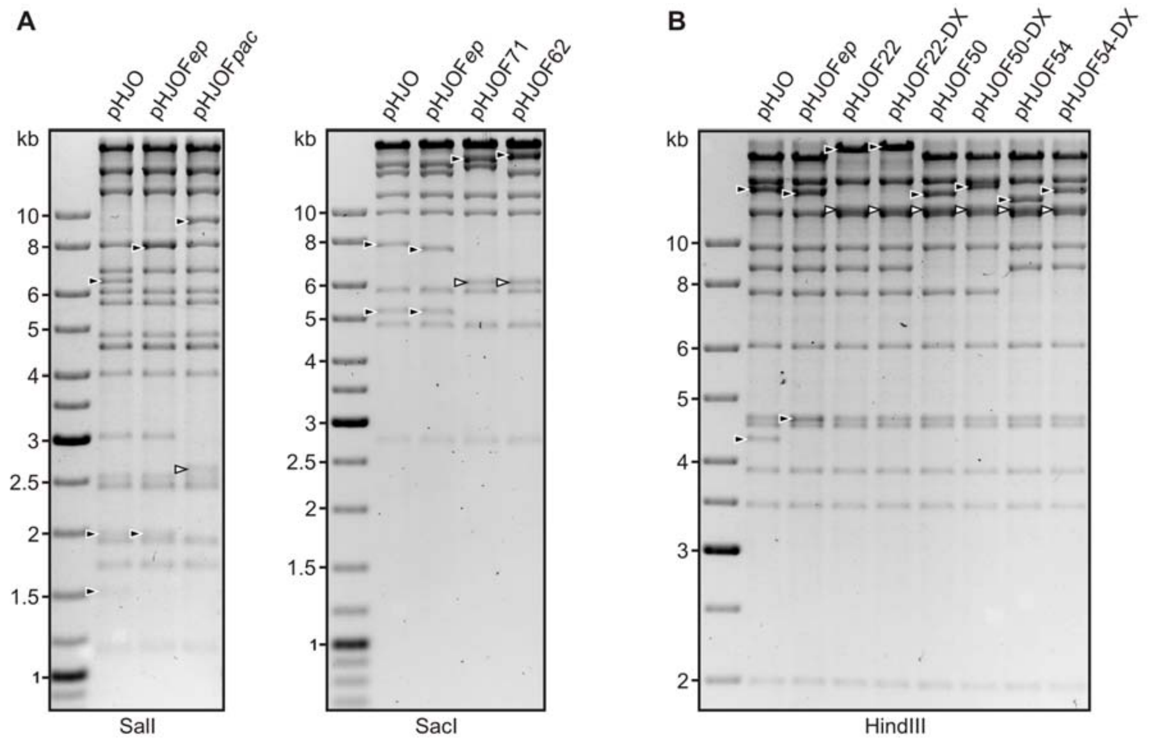

Figure S2. RFLP analysis of the mini-F vector transposition and insertion of genomic duplication. BAC DNA from *E. coli* was digested and the resulting DNA fragments were electrophoretically separated in 0.6% agarose gels and visualized by ethidium bromide staining. Fragments containing mini-F vector sequences (white framed black arrows) or the reconstituted unique AvrII site of the US region (black framed white arrows) are marked. **A)** SalI-RFLP analysis of pHJOF*pac* or SacI-RFLP analysis of pHJOF71 or pHJOF62 in comparison to original pHJO or pHJOF*ep*. **B)** HindIII-RFLP analysis of pHJOF22, pHJOF50, or pHJOF54, and the three pHJO-DX variants in comparison to pHJO, pHJOF*ep*. All rearrangements of the pHJO variants in A and B in comparison to original pHJO were as expected. A DNA ladder with indication of sizes in kilo base pairs (kb) is given.
