# Supplementary material for: Red-Mediated Transposition and Final Release of the Mini-F Vector of a Cloned Infectious Herpesvirus Genome
Source: PLoS One. 2009 Dec 4;4(12):e8178. doi: 10.1371/journal.pone.0008178 (PMC2780728; doi:10.1371/journal.pone.0008178)
Supplement: Figure S3 — Genome-intrinsic mini-F vector release from ORF62/71. (0.05 MB PDF) [file pone.0008178.s003.pdf]

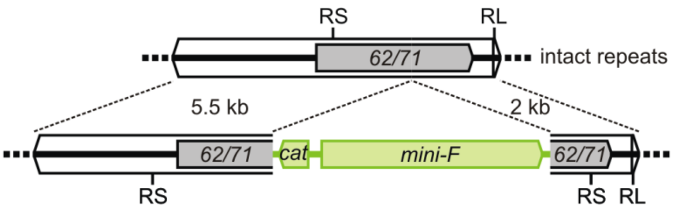

Figure S3. Genome-intrinsic mini-F vector release from *ORF62/71*. A) Putative required recombination events to release the mini-F vector sequences (green) in pHJOF62- or pHJOF71-derived virus. The 5.5 kb and 2 kb fragments of the RS regions flanking the mini-F cassette may recombine with intact inverted repeats of concatemers or of other replicating genomes to release the vector elements.
