# Supplementary material for: Red-Mediated Transposition and Final Release of the Mini-F Vector of a Cloned Infectious Herpesvirus Genome
Source: PLoS One. 2009 Dec 4;4(12):e8178. doi: 10.1371/journal.pone.0008178 (PMC2780728; doi:10.1371/journal.pone.0008178)
Supplement: Table S1 — Detection (A), Cloning (B) and En passant (C) primers used in this study. (0.01 MB PDF) [file pone.0008178.s004.pdf]

A) Detection

Primer	Sequence (5´- 3´)	Primer	Sequence (5´- 3´)
**P1**	CTTCGACCCGTCTTCCTTCC	**P3**	TGCCACTCATCGCAGTACTG
**P2**	AGGTGTTCGGGAATCTGATAGC	**P4**	AGGCATTTCAGTCAGTTGCTC

B) Cloning

Primer	Sequence (5´- 3´)	Primer	Sequence (5´- 3´)
**A**	TACGCTCGAGGATCCTACTCGAGGTCA	**I**	ACTAGTTATCGTTCTCGCCCATTATACG
**B**	TGACCTCGAGTAGGATCCTCGAGCGTA	**J**	ACTAGTTTTCCGCTAATTAAATAAACGGAG
**C**	TACGCTCGAGCATGCTACTCGAGGTC	**K**	GACTAGTTACACGTGATACTGAGACAAAGCG
**D**	GACCTCGAGTAGCATGCTCGAGCGTA	**L**	GACTAGTGGGAGGATGAGGCTGTGAGA
**E**	ACTAGTTATGTGTGCTCTTGTTGATCTCCC	**M**	CGCGTCGACCCTGCCGCAGAACCACTCGTGC
**F**	ACTAGTAACTTGTAACGCGTATAAGTTCCG	**N**	CGCGTCGACCCTAGGGGGCCCCCCTCCCGGGG
**G**	ACTAGTATTGTGGGGGAGTATAATTGTCC	**O**	GACTAGTGGATCCAGGGGGGCCCAGCCCTCTC
**H**	ACTAGTATTAACACGTGTATATAATGCGCC	**P**	GACTAGTCGTCCGCGCAACTGAAAATG

C) *En passant*

Primer	Sequence (5´- 3´)
**Q**	ATAAATACCTGTGACGGAAGATCACTTCGCAGAATAAATAAATCCCGATTTATTCAACAAAGCCACGT
**R**	TTACGTTATTATTAAATAAAACATGTAGACATTATTAATAATCCTAGGAACAATCAAATCCATATTTGTAA[illegible] TTATGTTTAACCCCTCCCTAGGGATAACAGGGTAATGCCAG

Table S1: Detection (A), Cloning (B) and *En passant* (C) primers used in this study.
